# The Prevalence and Causes of Primary Infertility in Iran: A Population-Based Study

**DOI:** 10.5539/gjhs.v7n6p226

**Published:** 2015-04-16

**Authors:** Hadigheh Kazemijaliseh, Fahimeh Ramezani Tehrani, Samira Behboudi-Gandevani, Farhad Hosseinpanah, Davood Khalili, Fereidoun Azizi

**Affiliations:** 1 Reproductive Endocrinology Research Center, Research Institute for Endocrine Sciences, Shahid Beheshti University of Medical Sciences, Tehran, Iran; 2 Obesity Research Center, Research Institute for Endocrine Sciences, Shahid Beheshti Universityof Medical Sciences, Tehran, Iran; 3 Prevention of Metabolic Disorders Research Center, Research Institute for Endocrine Sciences, Shahid Beheshti University of Medical Sciences, Tehran, Iran; 4 Endocrine Research Center, Research Institute for Endocrine Sciences, Shahid Beheshti University of Medical Sciences, Tehran, Iran

**Keywords:** Iran, primary infertility, prevalence, population-based study

## Abstract

**Background:**

Primary infertility is a health issue among women over the world. The aim of this study was to investigate the prevalence and causes of primary infertility based on a population-based study in an urban area of Iran.

**Materials and Methods:**

In a cross-sectional study, a total of 1067 married women who participated in the Tehran Lipid and Glucose Study were randomly selected using systematic random sampling. Unmarried women, those with unwilling pregnancy and duration of marriage below one year were excluded from the study. Data was collected by using validated ad-hoc questionnaires. Descriptive and inferential statistics were used for data analysis.

**Results:**

The mean (SD) of age and marriage age of the studied women were 40.3 (9.3) and 20.6 (4.49) years, respectively; the overall prevalence of lifetime primary infertility among couples was 17.3% (185/1067). Ovulatory disorder (39.7%) and male factors (29.1%) were the main causes of primary infertility. In addition, 31 (17%) of the women were diagnosed with more than one cause. According to the logistic regression analysis, primary infertility was independently related to the old age of women (OR: 1.37; 95% CI: 1.14–13.63, P.value: 0.001), higher BMI (OR: 1.95; 95% CI: 1.87–4.14, P.value: 0.003), active smoking (OR: 1.47; 95% CI: 1.38–3.53, P.value: 0.012) and higher educational level (OR: 2.23; 95% CI: 1.12–5.53, P.value: 0.03).

**Conclusion:**

The prevalence of primary infertility in Iran was higher than the worldwide trends of infertility, indicating that understanding such risks help healthcare providers and policy makers to design and implement interventions to slow down this trend.

## 1. Introduction

Infertility is a common problem affecting one couple out of every six couples (Brugo-Olmedo, Chillik, & Kopelman, 2001). It is defined as incapacity to become pregnant after mostly 12 months of sexual intercourse, without using any contraception. In this respect, primary infertility was defined as the “Inability to conceive within one year of exposure to pregnancy (i.e.-being sexually active, usingnon-contraception, and non-lactating) among women aged 15-49 years” (Nygren et al., 2011).

The 12-month prevalence rate of infertility ranges from 3.5% to 16.7% in more developed countries and from 6.9% to 9.3% in the less-developed ones, with an estimated overall median prevalence of 9% (Boivin, Bunting, Collins & Nygren, 2007). Differences between the developed and developing world are emerging due to variations of infertility care and different socio-cultural values surrounding procreation and childlessness (OMbelet, 2011). In recent years, the prevalence of infertility has been significantly increased (Brugo-Olmed et al., 2001). The increasing trend could be due to delayed childbearing of couples, alterations in semen quality due to habits such as cigarette smoking and alcohol, changes in sexual behavior and elimination of most taboos (Brugo-Olmed et al., 2001).

Infertility has been recognized as a potentially serious, costly and burdensome problem for affected families (Mohammad & Ardalan, 2009). It is a medical circumstance that not only has health implications for those involved, but also is a condition linked to individual human rights (OMbelet, 2011; Naab, Brown & Heidrich, 2013). The social stigma of childlessness still leads to isolation and abandonment in many developing countries (Gannon, Glover, & Abel, 2004).

There are many biological causes of infertility such as ovulatory factors, utero-tubal peritoneal factor, semen migration factor, and the male factor that are present in 20%, 30%, 10%, and 30% of couples, respectively (Meng et al., 2014; Zargar, Wani, Masoodi, Laway & Salahuddin, 1997). Around 40% of all infertile couples exhibit a combination of factors and about 15% of them may not display any objective alteration leading to a definite diagnosis (Aziz, Agarwal, Nallella & Thomas, 2006). However, the main challenge in generating global estimates in the prevalence and different causes of infertility are the scarcity of population-based studies. Therefore, accurate assessment of the prevalence of infertility using epidemiological studies are required in order to plan appropriate strategies for prevention, treatment and management of infertility and its socio-economic consequences. Therefore, this population-based study aimed to identify the prevalence and cause of primary infertility in a sample of reproductive-aged women in an urban area of Iran.

## 2. Methods

**Figure 1:**
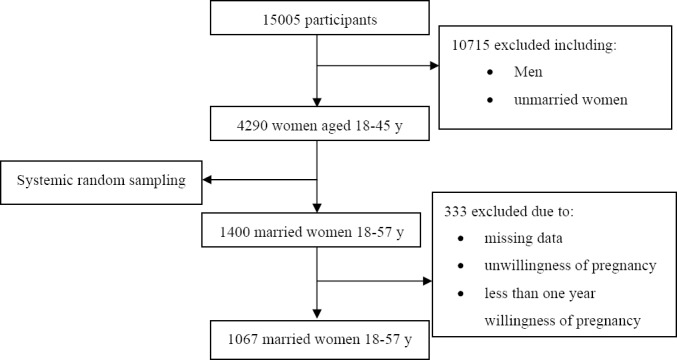
Study flowchart

The women were selected from the participants of the Tehran Lipid and Glucose Study (TLGS), as an ongoing prospective population-based cohort study initiated in 1998 in order to explore the prevalence and risk factors of non-communicable diseases (Azizi et al., 2003). After obtaining the informed consent, 15005 ethnic Iranian residents aged > 3 y, of district 13 of Tehran city, were recruited and followed up at three year intervals; 4290 women, aged 18-45 years participated in the TLGS. For the present study, 1400 women were randomly selected using the systematic random sampling method. All unmarried women, those with unwilling pregnancy and duration of marriage below one year were excluded from the study. Figure 1 shows the process of the study.

Data was collected using researcher-made questionnaires to obtain information on demographic characteristics, general medical, menstrual, reproduction, and contraception histories of couples.

The content validity of the questionnaire was assessed by 15 gynecologists and reproductive health experts. The reliability of the instrument was determined using test-retest to determine the level of agreement between responses in a10 days-interval (r=0.91). Internal consistency was measured using Cronbach’s alpha correlation coefficient (α=0.79).

According to the WHO manual for the standardized investigation and diagnosis of the infertile couple, the causes of infertility were classified to male factor, tubal ovulation, and endocrine disorders (Rowe, Comhaire, Hargreave & Mellows, 1993).

In this study, lifetime primary infertility was defined as the failure to achieve a clinical pregnancy after 12 months or more of regular unprotected sexual intercourse whether or not having a child (Nygren et al., 2011).

The ethical review board of the Shahid Beheshti University of Medical Sciences approved the study proposal. Study aims and methods were explained to potential participants, who were assured of their anonymity throughout the study and that they were free to withdraw at any time. Lastly, written informed consent was obtained from the participants.

Data analysis was conducted via the Statistical Package for the Social Sciences version 11 (SPSS Inc., Chicago, IL, USA). Continuous measures shown as mean ± standard deviation (SD), and categorical variables were expressed as percentages. For comparison of variables *t*-test, Chi-square and ANOVA test were used; 95% confidence interval was considered for the estimation of prevalence rate of primary infertility. Multiple logistic regression tests were used to find the relationship of independent factors associated with the dual dependent variable of primary infertility. Statistical tests were two-sided and P < 0.05 was considered statistically significant.

## 3. Results

Of the 1400 women interviewed, 333 (23.7%) were excluded from analysis, for various reasons described earlier (Figure 1).

The baseline socio-demographic and obstetric characteristics of the women are shown in Table 1. The mean (SD) age and marriage age of the studied women were 40.3 (9.3) and 20.6 (4.49) years, respectively.

**Table 1:** Socio-demographic and obstetric characteristics of participants (N= 1067) and comparison between them by groups

	Baseline	Infertile women n= 185	Fertile women n= 882	p-value
Age of female (yrs), mean (SD) n (%)	40.3 (9.3)	40.3 (5.2)	41.6 (8.6)	0.063
Marriage age, (yrs), mean (SD)	20.6 (4.49)	20.9 (4.29)	20.5 (5.3)	0.269
BMI (kg/m2), mean (SD)	26.6 (12.3)	26.57 (5.70)	26.8 (4.5)	0.021
Education (yrs)	10.1 (4.3)	11.8 (5.4)	8.3 (3.27)*	0.001
Current main activity, n (%)				
Unemployed	669 (62.6)	121 (65.4)	548 (62.1)	0.087
Employed	398 (37.3)	64 (34.5)	334 (37.8)	0.151
Smoking history^#^ n (%)				
Never	1041 (97.5)	169 (91.3)	872 (98.8)	0.075
Past	15 (1.4)	8 (4.3)	7 (0.7)*	0.031
Current	11(1.3)	8 (4.3)	3 (0.3)*	0.042

# Smoking status was defined as current smoking, having smoked and still smoking during last one month; past smoking, having smoked, but have not smoked during last one month; and never smoking.

Comparisons of characteristics of infertile and fertile women showed that infertile women had higher educational level and BMI (P = 0.001) and (P = 0.021), respectively, (Table 1).

In this respect, although the total prevalence of smoking history in the women was generally low (2.5%), but infertile women had significantly more positive smoking history than fertile women (Table 1).

The overall prevalence of life time primary infertility among the couples was 17.3% (185/1067).Distribution of the causes of infertility in the participants is presented in Table 2.

**Table 2:** Distribution of the causes of infertility among the study women#*

Diagnosis	No. of cases (%)
Ovulatory disorder	73 (39.7)
Tubal disorder	7 (3.7)
Male factors	54 (29.1)
Unexplained	27 (14.4)
Endometriosis	15 (8.2)
Other	9 (4.7)

# The percentages were rounded to the nearest unit; their summay therefore not amount to 100%.

* 31 (17%) of participants had more than one diagnosis

Ovulatory disorder (39.7%) and male factors (29.1%) were the main causes of primary infertility in our participants; in addition, 31 (17%) of the participants were diagnosed with more than one cause.

In this respect, logistic regression analysis showed that primary infertility was independently related to the old age of women (OR: 1.37; 95% CI: 1.14–13.63, P.value: 0.001), higher BMI (OR: 1.95; 95% CI: 1.87–4.14, P.value: 0.003), active smoking (OR: 1.47; 95% CI: 1.38–3.53, P.value: 0.012) and higher educational level (OR: 2.23; 95% CI: 1.12–5.53, P.value: 0.03).

Also, 82% of infertile couples sought help to solve their problem from the gynecologists, urologists and consulted healthcare professionals. Over half of the couples, 108/185 (58.3%), who received treatment including ovulation induction, IVF/ICSI, donor eggs or surrogate uterus, weight loss, and intrauterine insemination, became pregnant within 1 year of treatments; 12.4%, of the treatments were unsuccessful and 4.8% (9/185) of the couples withdrew the treatment completely.

## 4. Discussion

Infertility is a prevalent condition that has profound socio-economic and health consequences on both the individual and society. Despite the important consequences of infertility, estimations of its prevalence are limited. The reported prevalence of infertility ranges between 3.5%-22% in different countries, due possibly to the recruitment process of the study population, infertility definitions and method of estimation (Larsen, 2005). Furthermore, advances in the diagnosis, treatment and prevention of infertility in the past decades have led to considerable changes in the worldwide prevalence of infertility.

In our population-based study, the overall prevalence of lifetime primary infertility among the participants was 17.3% demonstrating a high prevalence of primary infertility in Iranian women of reproductive age.

Prevalence of primary infertility in this study was comparable to the lifetime primary infertility rate estimated by the National Infertility Survey (2004–2005) and the Tehran Study (1997), which was 24.1% and 21.9%, respectively (Vahidi, Ardalan, & Mohammad, 2009; Noorbala & Mohammad, 2001). Also, Esmaeilzadeh et al. (2012) in a population-based study in Babol, Iran, studied the prevalence of infertility and self-reported cause of infertility. Among clustered sample of 1,140 women aged 20-45 years, 15.5% of the women experienced difficulty conceiving at some stage in their lives. In their study, the prevalence of primary infertility was 4.3% (CI: 2.3, 6.3), which is lower than the rate reported in our study. This discrepancy is due to the fact that Esmaeilzadeh et al.’s study reported the prevalence of current primary infertility, but we reported life time primary infertility. In agreement with our study’s findings, the most frequently self-reported cause of infertility in this study was ovulation problem (39.2% vs. 39.7). In addition, Rostami Dovom et al. (2014) in a population-based study of selected provinces of Iran reported the overall prevalence of lifetime infertility to be 21.1% (Rostami Dovom et al., 2014); our prevalence however is similar to that of industrialized countries estimated to be around 15% (Organization WH, 1991); and is higher than global trends of infertility that has been reported to be 10.5% (Mascarenhas, Flaxman, Boerma, Vanderpoel & Stevens, 2012). This pattern may be attributed to the consequences of untreated reproductive tract infections, including both STIs such as Neisseria gonorrhoeae and Chlamydia trachomatis, and, to a lesser extent, to infections from unsafe abortions. In addition to different socio-economic conditions and ethnicities, and lack of uniformity in methods of calculating primary infertility rates hamper comparative studies between populations (Rostami Dovom et al., 2014).

Additional analysis showed that the ovulatory disorders are common problems leading to primary infertility in Iran, which could be due to older ages of marriage for women and tendencies of delayed childbearing among couples (Behboudi-Gandevani, Ziaei, Khalajabadi-Farahani, & Jasper, 2013). It is reported that aging is one of the main reasons of infertility in the women (Szafarowska & Jerzak, 2013).

Our study showed that help seeking behaviors for infertility services is common in the Iranian population. More than 75% who meet criteria for fertility problems seek medical help, which in itself is a complex family health issue and inherently includes several issues with social dimensions. Stephen et al. in the United States showed that around 42% of infertile women had sought some form of infertility services (Stephen & Chandra, 2000). This behavior is lower than infertility service uses in Iran. It may be due to the current notion that having children in Iran is the only way, for women to enhance their status in the family and community (Hasanpoor-Azghdy, Simbar, & Vedadhir, 2014).

We found that infertility was increased by aging, higher BMI, active smoking and higher educational levels. In agreement to our study, Skirbekk et al. reported that in educated women, delayed marriage and postponement of pregnancy may increase risk of infertility (Skirbekk, 2008). Dechanet et al. reported that obesity and cigarette smoking were factors associated with decreased fertility by causing delay in conception and decreased IVF results (Dechanet, Belaisch-Allart, & Hédon, 2010).

The data in this study were extracted from the population-based TLGS study, which was a complete record of an individual’s health care over time. Some limitations in the present study could affect the estimated rate of primary infertility, should be addressed. The estimation of infertility was based on information of self-report questionnaire; however, some women might not engage regularly in sexual intercourse and have a lower chance of having a child. Also, our assessment may be underestimated, because most of the couples in Iran used modern contraception methods before their first child (Abbasi-Shavazi, McDonald, & Hosseini-Chavoshi, 2009).

Despite extensive data seeking, we relied on women’s reported couple status, births, contraceptive use, and the desire for a child; these assumptions may be inaccurate, as women may not report accurately on this sensitive topic, also, infertile women may state that they do not want a child as a coping mechanism (Larsen, 2005).

## 5. Conclusion

The prevalence of primary infertility in Iran was higher than global trends of infertility, with the ovulatory disorder being the main cause of primary infertility in Iran. Understanding the risks is important for women to help them to make informed decisions on the timing of conception and also for reproductive healthcare providers and policy makers to design and provide appropriate implement intervention to slow down this trend.
